# Immunocompetent Mice Infected by Two Lineages of Dengue Virus Type 2: Observations on the Pathology of the Lung, Heart and Skeletal Muscle

**DOI:** 10.3390/microorganisms9122536

**Published:** 2021-12-08

**Authors:** Fernanda Cunha Jácome, Gabriela Cardoso Caldas, Arthur da Costa Rasinhas, Ana Luisa Teixeira de Almeida, Daniel Dias Coutinho de Souza, Amanda Carlos Paulino, Marcos Alexandre Nunes da Silva, Derick Mendes Bandeira, Ortrud Monika Barth, Flavia Barreto dos Santos, Debora Ferreira Barreto-Vieira

**Affiliations:** 1 Laboratory of Viral Morphology and Morphogenesis, Instituto Oswaldo Cruz, Fiocruz, Avenida Brasil 4365, Rio de Janeiro 21040-900, Brazil; gabrielacardosocaldas@gmail.com (G.C.C.); rasinhas@protonmail.com (A.d.C.R.); almeida.analuisa98@gmail.com (A.L.T.d.A.); dcoutinho@id.uff.br (D.D.C.d.S.); amandacarlos.bio@gmail.com (A.C.P.); marquinhosans@hotmail.com (M.A.N.d.S.); derick_mendes@live.com (D.M.B.); monikabarth@gmail.com (O.M.B.); barreto@ioc.fiocruz.br (D.F.B.-V.); 2 Laboratory of Viral Immunology, Instituto Oswaldo Cruz, Fiocruz, Avenida Brasil 4365, Rio de Janeiro 21040-900, Brazil; flaviabarretod1@gmail.com

**Keywords:** dengue virus serotype 2, lineages, BALB/c mice, lung, heart, skeletal muscle

## Abstract

Dengue virus (DENV) infection by one of the four serotypes (DENV-1 to 4) may result in a wide spectrum of clinical manifestations, with unpredictable evolution and organ involvement. Due to its association with severe epidemics and clinical manifestations, DENV-2 has been substantially investigated. In fact, the first emergence of a new lineage of the DENV-2 Asian/American genotype in Brazil (Lineage II) in 2008 was associated with severe cases and increased mortality related to organ involvement. A major challenge for dengue pathogenesis studies has been a suitable animal model, but the use of immune-competent mice, although sometimes controversial, has proven to be useful, as histological observations in infected animals reveal tissue alterations consistent to those observed in dengue human cases. Here, we aimed to investigate the outcomes caused by two distinct lineages of the DENV-2 Asian/American genotype in the lung, heart and skeletal muscle tissues of infected BALB/c mice. Tissues were submitted to histopathology, immunohistochemistry, histomorphometry and transmission electron microscopy (TEM) analysis. The viral genome was detected in heart and skeletal muscle samples. The viral antigen was detected in cardiomyocytes and endothelial cells of heart tissue. Heart and lung tissue samples presented morphological alterations comparable to those seen in dengue human cases. Creatine kinase serum levels were higher in mice infected with both lineages of DENV-2. Additionally, statistically significant differences, concerning alveolar septa thickening and heart weight, were observed between BALB/c mice infected with both DENV-2 lineages, which was demonstrated to be an appropriate experimental model for dengue pathogenesis studies on lung, heart and skeletal muscle tissues.

## 1. Introduction

The incidence of dengue has grown dramatically around the world in recent decades, and currently, more than half of the global population lives in areas with a risk of DENV transmission [1,2]. Infection by any of the four serotypes may result in a wide spectrum of clinical manifestations with unpredictable evolution and outcome, varying from self-limited flu-like illnesses to a severe form of the disease, characterized by thrombocytopenia, coagulopathy, increased vascular permeability that may lead to multiple organ impairment, hemorrhage, hypovolemic shock and death [3,4,5,6]. Moreover, during infection, organ involvement such as hepatic, cardiac, renal, pulmonary, neurological, muscular, splenic, gastrointestinal and even ocular injury can be observed [5,7,8,9].

Heart, lung and skeletal muscle are not considered the main DENV targets. Regarding the lung, a study has suggested that pulmonary involvement during DENV infection is mild to moderate and is more likely to be observed in patients presenting more severe symptoms [10]. Respiratory complications vary from dyspnea to acute respiratory failure and/or acute respiratory distress syndrome [11]. Since its first report in 1926 [12], cardiac involvement during dengue has been increasingly reported and it is mostly associated with severe cases [13,14,15,16,17,18]. Several arboviruses have tropism for muscle cells and can cause pathological alterations of the skeletal muscle [19]. Myalgia is a common feature in dengue patients and muscle pain can persist beyond the acute phase of dengue [20]. The disruption of skeletal muscle integrity leads to the release of intracellular muscle components, such as CK into the bloodstream and extracellular space [21], and a study showed an increase in the levels of this protein in dengue patient sera [22]. Moreover, some studies have reported muscle alterations in human samples [22,23,24,25,26,27].

The pathophysiological mechanisms involved in severe cases are still to be fully elucidated [28] but studies do consider viral strains’ virulence [5,29]. DENV-2 has traditionally been the most studied serotype due to its association with large epidemics and severe clinical manifestations [30]. Moreover, the DENV-2 Asian/American genotype, introduced in Brazil in the 1990s and currently circulating in the country [31,32,33,34], has been associated with a higher fitness in both humans and mosquitoes [35]. The emergence of a new lineage (Lineage II) of the DENV-2 Asian/American genotype in 2008, different than that introduced in the 1990s (Lineage I), was associated with increased pathogenicity, reflected by the high number of severe cases, hospitalizations and deaths in Brazil [30,36,37]. Replacements of DENV-2 lineages over outbreaks have been described as a common phenomenon in American countries [33].

The establishment of experimental models for DENV infection studies and their impact on disease severity is of particular relevance. Immunocompetent mice, including BALB/c, have been used for DENV pathogenesis and tropism studies by using different infection routes, and clinical signs as well as tissue alterations similar to those observed in dengue human cases were observed, even using epidemic non-adapted DENV strains. Viral genomes and antigens have been detected in the spleen, liver, brain, heart, lung, kidney and saliva [38,39,40,41,42,43,44,45,46,47,48,49,50,51]. Moreover, BALB/c was recently shown to be useful in a therapeutic approach to treat DENV infection with improved tissue repair and regeneration [52].

We previously showed the BALB/c susceptibility to Lineages I and II of DENV-2 of the Asian/American genotype and its impact in the kidney and liver [47,48]; however, considering the systemic profile DENV infections may present, here, we aimed to further investigate the impact of those lineages in the lung, heart and skeletal muscle tissues of infected mice.

## 2. Materials and Methods

### 2.1. Ethical Statements

All procedures performed in this study were approved by the Animal Ethic Committee (protocol L-023/2018) and the Human Research Ethics Committee (protocol 274/05) of Oswaldo Cruz Institute (IOC), Oswaldo Cruz Foundation (FIOCRUZ).

### 2.2. Viral Strains

Strains BR/RJ66985/2000 and BR/RJ0337/2008 (GenBank #HQ012518 and #HQ01253, respectively), representative of Lineages I and II of the DENV-2 Asian/American genotype [32], were isolated from patient sera at Flavivirus Laboratory, IOC, FIOCRUZ, during the epidemics of 2000 and 2008, respectively, and kindly provided. The DENV-2-infecting serotype was confirmed by indirect immunofluorescence, using DENV type-specific monoclonal antibody (3H5) and RT-PCR [53,54]. Virus stock was prepared by inoculating 100 µL of each strain into cell culture bottles containing the mosquito *Aedes albopictus* C6/36 cell line at a concentration of 5 × 10^5^ cells/mL. Titers (BR/RJ66985/2000: 10^6.66^ TCID_50_/1 mL and BR/RJ0337/2008: 10^9^ TCID_50_/1 mL) were calculated by the Reed–Muench method [55]. The viruses did not undergo any passages through mouse brain for neuroadaptation.

### 2.3. Study Design

For experimental viral infection, BALB/c mice were inoculated by the intravenous route (IV) through the caudal vein. Inocula volume was 100 µL and viral concentration was 10,000 TCID_50_/0.1 mL. The mice were anesthetized (0.2 mL of ketamine, xylazine and tramadol solution) and euthanized 24, 48 or 72 h post-infection (hpi) or 7 or 14 days post-infection (dpi), according to their experimental group.

Histopathological, morphometric and ultrastructural analysis and viral genome and antigen detection were performed with organ samples of mice euthanized at 72 hpi. Lung, heart and skeletal muscle samples destined for morphological analysis and immunohistochemistry assay were fixed with proper fixatives. Those destined for qRT-PCR assay were stored at −80 °C. Mice lungs and hearts were weighted immediately after harvesting (72 hpi, 7 dpi and 14 dpi). Noninfected mice were used as negative controls. Table 1 shows the number of mice used in this study.

### 2.4. Histopathology

For each DENV-2 lineage, 10 mice were infected. At 72 hpi, the mice were euthanized, and lung, heart and skeletal muscle samples were collected and fixed in Millonig buffered formalin. The samples were then dehydrated in decreasing concentrations of ethanol, clarified in xylene and embedded in paraffin. Tissue sections 5 µm thick were obtained using a microtome (Leica 2025, Wetzlar, Germany), stained with hematoxylin and eosin (H&E) and analyzed using a bright-field microscope (AxioHome, Carl Zeiss, Oberkochen, Germany). Five noninfected mice were used as a negative control. All procedures were carried out in collaboration with Pathology Laboratory, IOC, FIOCRUZ.

### 2.5. Immunohistochemistry

For immunohistochemistry assays, five slides containing histological sections of lung, heart and skeletal muscle of BALB/c mice euthanized at 72 hpi were selected. The slides were heated to 60 °C and immersed in xylene for removal of paraffin. The samples were then dehydrated in decreasing concentrations of ethanol, prior to antigen retrieval, and immersed in Dako buffer in a pressure cooker. After cooling, the sections were incubated with anti-4G2 antibody produced in rabbit (1:200), used as the primary antibody, and later with the secondary anti-rabbit antibody conjugated with horseradish peroxidase (Spring Bioscience, Pleasanton, CA, USA). Finally, the slides were counterstained with Harris hematoxylin and analyzed under a bright-field microscope (AxioHome, Carl Zeiss, Oberkochen, Germany). Slides containing histological sections from noninfected mice were used as a negative control.

### 2.6. Transmission Electron Microscopy (TEM)

For ultrastructural studies, organ samples from 15 mice (five from the control group and five from the groups infected with both DENV2 strains) euthanized at 72 hpi were processed as described by Barreto-Vieira [56]. Briefly, samples were fixed by immersion in glutaraldehyde (Electron Microscopy Sciences, Hatfield, PA, USA) at 2% diluted in sodium cacodylate buffer (0.2 M, pH 7.2), cleaved into smaller fragments (~1 mm^3^), post-fixed in 1% osmium tetroxide and dehydrated in increasing concentrations of acetone. Subsequently, the samples were embedded in epoxy resin (Electron Microscopy Sciences). Ultra-thin sections (50–70 nm thick) were obtained with the aid of an ultramicrotome (Leica, Wetzlar, Germany), placed on copper grids and counterstained with uranyl acetate and lead citrate. Finally, the samples were observed under the Hitachi HT 7800 TEM (Hitachi, Tokyo, Japan).

### 2.7. Histomorphometry

To perform the histomorphometric analysis, a total of 15 slides containing histological sections of lungs from mice euthanized at 72 hpi stained with H&E were used. For each slide, 20 images of random areas were captured with the aid of a camera coupled to the AxioHome bright-field microscope (Carl Zeiss, Oberkochen, Germany) using a 40× objective lens for images, resulting in 300 areas. The analysis was performed using the public domain image processing program Image J. For each lung image, the thickness of 20 alveolar septa was measured. Alveolar septal thickness values were compiled for the experimental group and a simple mean was calculated.

### 2.8. CK Levels Analysis

For each DENV-2 lineage, 15 mice were infected. The mice were divided into three groups of five animals and each group was euthanized at different times after infection (24, 48 or 72 hpi). After the determined periods of infection, the mice were anesthetized, and blood was collected by cardiac puncture. The samples were then centrifuged for 10 min, at 5000 rotations per minute, to separate the serum from the cellular components. Noninfected mice (*n* = 5) blood was collected on the same day as the 72 hpi group. Blood levels of CK were measured by dry chemistry testing using the Vitros 250 equipment (Ortho Clinical, Jonhson & Jonhson) in collaboration with Instituto de Ciências e Tecnologia de Biomodelos (ICTB), FIOCRUZ. The assay was carried out immediately after sampling.

### 2.9. Viral Genome Extraction and Quantitation

For the extraction of the viral genome, lung, heart and skeletal muscle fragments from 10 mice infected with DENV-2 lineages euthanized at 72 hpi were macerated in 500 µL of Leibovitz culture medium (Invitrogen, Waltham, MA, USA) and centrifuged for 15 min at 10,000 RPM at a temperature of 4 °C. RNA was extracted from 140 µL of the macerated organ supernatant using the QIAmp Viral RNA mini kit (Qiagen, Düsseldorf, Germany) according to the manufacturer’s recommendations. For the detection and quantification of the viral genome, a standard curve was constructed from a serial dilution of RNA extracted from a DENV-2 sample (strain S16083), with a known titer (8.7 × 10^6^ PFU/mL). The protocol used was described by Johnson et al. [57], using the primers DENJ2-R (5′-CCATCTGCAGCAACACCATCTC-3′) and DENJ2-F (5′-CAGGTTATGGCACTGTCACGAT-3′), designed from a fragment of the 3′ non-coding region, and the probe DENJ2-P (CY5 5′-CTCTCCGAGAACAGGCCTCGACTTCAA-3′ BHQ-1). The reaction was performed according to the protocol of the commercial kit SuperScript III Platinum One-Step Quantitative RT-PCR (Invitrogen, USA), with ideal concentrations of the primers and probe determined by optimization assays. In a 96-microwell optical-bottom plate Waltham, MA, USA1 µL of each primer (50 µM) and 12.5 µL of the reaction mix (0.4 µM of each dNTP and 6 µM of MgSO_4_) were added, followed by 0.5 µL SuperScript III RT enzyme, 3.5 µL DNase/RNase free water, 1 µL MgSO_4_ (5 mM) and 0.75 µL probe (9 µM), for a total volume of 20 µL per well. Soon after, 5 mL of extracted RNA was added, obtaining a final volume of 25 mL/reaction. Each sample and control were applied in duplicate. The plates were placed on the 7500 FAST platform (Applied Biosystems, USA) for the qRT-PCR reaction, according to the following cycling parameters: reverse transcription (50 °C, 15′, 1 cycle), enzyme activation (95 °C, 2′, 1 cycle), denaturation (95 °C, 15″, 40 cycles) and annealing/extension (60 °C, 1′, 40 cycles).

### 2.10. Statistical Analisys

A database was constructed with the data collected during the experiment. *T*-tests were performed using SPSS 25 software (IBM) and graphics were constructed using GraphPad Prism 8.0.1 software. *p* values of *p* ≤ 0.05 were considered statistically significant.

## 3. Results

### 3.1. Organ Weight

A slight increase in the mean variation in lung weight of mice infected with DENV-2 Lineage I was observed when compared to noninfected mice (mean = 0.203 g) at 72 hpi and 7 dpi (means: 0.210 g and 0.214 g, respectively); however, the mean value decreased at 14 dpi (0.199 g). For animals infected with Lineage II, on the other hand, the variation in lung weight was different, as the mean of infected mice was greater than that of the negative control at all times of infection (means: 72 hpi = 0.217 g; 7 dpi = 0.223 g; 14 dpi = 0.233 g).

**Figure 1:**
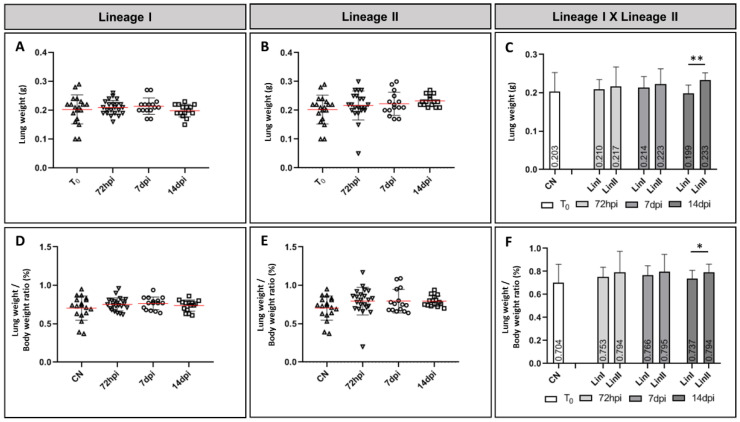
Mean lung weight (**A**–**C**) and lung weight/body weight ratio (%) (**D**–**F**) of BALB/c mice noninfected and infected with DENV-2 lineages at 72 hpi, 7 dpi and 14 dpi. CN: (*N* = 19); LinI: 72 hpi (*N* = 22), 7 dpi (*N* = 15), 14 dpi (*N* = 15); LinII: 72 hpi (*N* = 22), 7 dpi (*N* = 15), 14 dpi (*N* = 15). NC: negative control, T_0_: before infection, hpi: hours post-infection, dpi: days post-infection, Lin: lineage, *: *p* < 0.05, **: *p* < 0.01.

The proportion between lung weight and body weight, on average, was higher in infected mice when compared to the noninfected group (mean = 0.704%). When analyzing mice infected with Lineage I, we observed that, on average, the ratio between lung weight and body weight was slightly higher at 7 dpi (mean = 0.766%). In mice infected with Lineage II, although the means did not vary during the entire period of infection, they were all higher when compared to Lineage I. There was a statistically significant difference between the infected groups that were euthanized 14 dpi in relation to the total organ weight and the organ weight/body weight ratio (*p* < 0.001 and *p* = 0.035, respectively). Figure 1 shows the lung weight variation (A–C) and the lung weight/body weight ratio (D–F).

The variation in heart weight of mice infected with Lineages I and II of DENV-2 presented distinct profiles. The mean heart weight of mice infected with Lineage I increased at all times of infection (mean: 72 hpi = 0.166 g; 7 dpi = 0.173 g; 14 dpi = 0.183 g) when compared to the mean of the negative control (mean = 0.158 g). The mean heart weight of animals infected with the other strain was slightly lower than the mean of the noninfected group at 72 hpi (mean = 0.156 g). Finally, mice euthanized at 7 dpi and 14 dpi showed an increase in mean heart weight (means = 0.163 g and 0.183 g, respectively).

**Figure 2:**
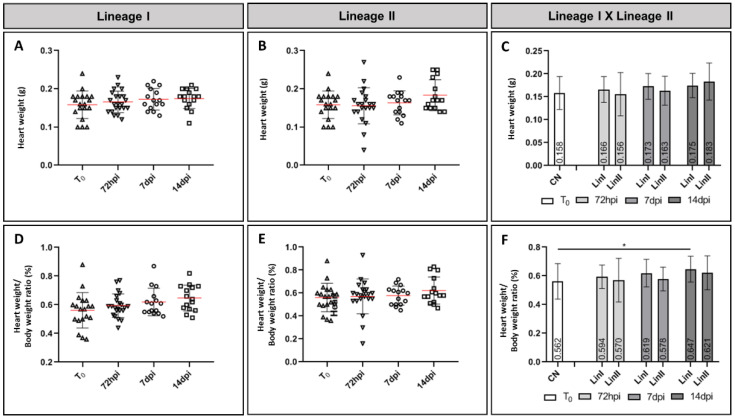
Mean heart weight (**A**–**C**) and heart weight/body weight ratio (%) (**D**–**F**) of BALB/c mice noninfected and infected with DENV-2 Lineages at 72 hpi, 7 dpi and 14 dpi. CN: (*N* = 19); LinI: 72 hpi (*N* = 22), 7 dpi (*N* = 15), 14 dpi (*N* = 15); LinII: 72 hpi (*N* = 22), 7 dpi (*N* = 15), 14 dpi (*N* = 15). NC: negative control, T_0_: before infection, hpi: hours post-infection, dpi: days post-infection, Lin: lineage, *: *p* < 0.05.

On average, the heart weight/body weight ratio of mice infected with DENV-2 lineages also increased compared to noninfected mice (mean = 0.562%). In mice infected with Lineages I and II, the ratio increased gradually, with a higher mean at 14 dpi (means = 0.647% and 0.621, respectively). The difference between the means of the control group and the group infected with Lineage I euthanized at 14 dpi was significant (*p* = 0.031). Figure 2 shows the heart weight variation (A–C) and heart weight/body weight ratio (D–F).

### 3.2. Morphology

**Figure 3:**
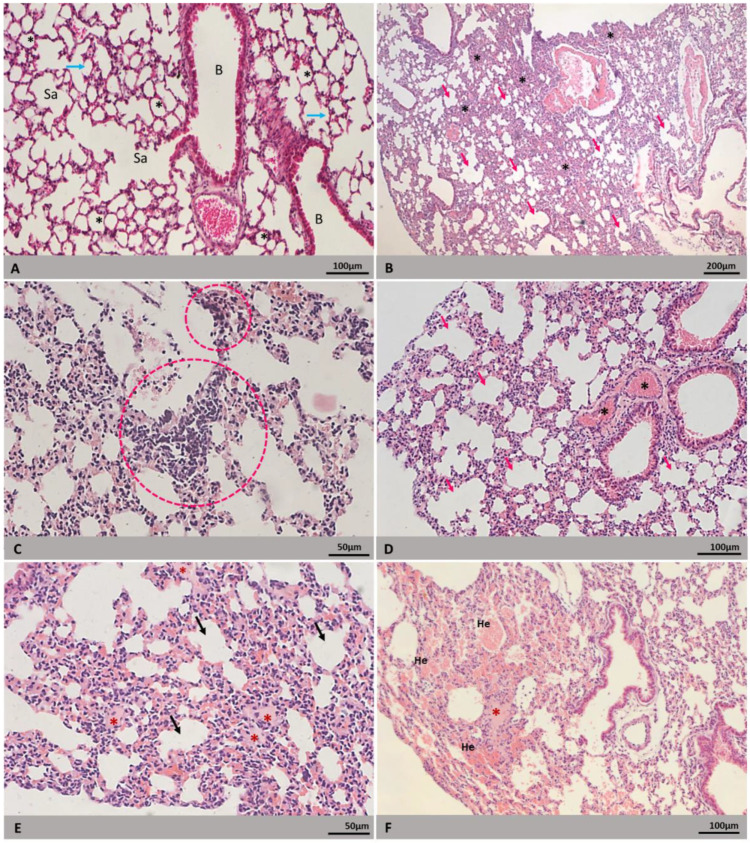
Histopathological alterations of lung of BALB/c mice. H&E staining. Euthanasia; 72 hpi. (**A**) Noninfected mouse. Alveolar sac (Sa), alveolar septum (arrow), alveolus (*), bronchiole (**B**). (**B**–**D**) Mice infected with DENV-2 lineages: (**B**) alveolar septa thickening (*), alveoli hyperinflation (arrow); (**C**) inflammatory infiltrate (circled area); (**D**) vascular congestion (*), alveoli hyperinflation (arrow); (**E**) alveolar edema (*), vascular congestion (arrow); (**F**) hemorrhage (He), edema (*). Experimental infection: (**B**,**C**,**E**) Lineage I, (**D**,**F**) Lineage II.

**Figure 4:**
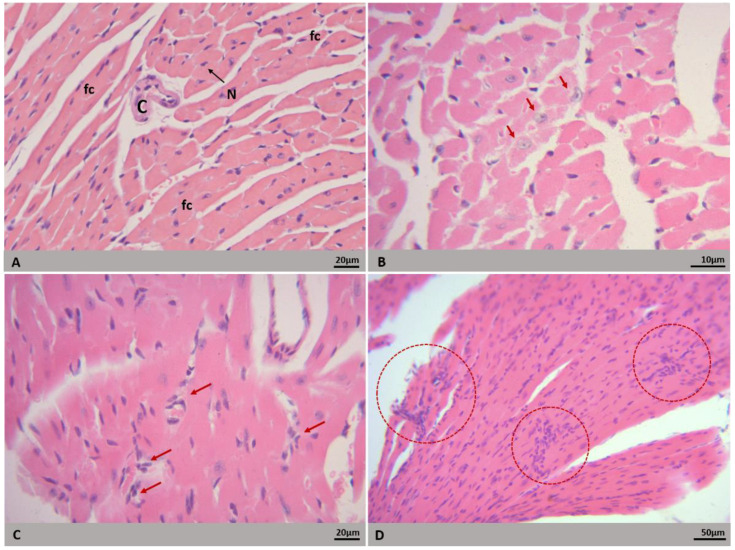
Histopathological alterations of heart of BALB/c mice. H&E staining. Euthanasia; 72 hpi. (**A**) Noninfected mouse. Cardiac fiber (fc), capillary (C), nucleus (N). (**B**–**D**) Mice infected with DENV-2 lineages: (**B**) rarefaction of cytoplasm and nuclear content (arrow); (**C**) inflammatory infiltrate (arrow); (**D**) increased interstitial cellularity. Experimental infection: (**C**) Lineage I, (**B**,**D**) Lineage II.

Lung tissues from noninfected BALB/c mice showed bronchioles, alveolar sacs, alveoli, no signs of rupture, edema, hemorrhage or other morphological changes (Figure 3A). Alveolar septa, however, presented some areas of mild thickening. The histopathological changes induced by the infection of the two DENV-2 lineages in the lung of BALB/c mice were focal and similar. The thickening of the alveolar septum (Figure 3B,D) and collapse of the alveolar space, due to the migration of inflammatory cells to the interstitium (Figure 3C), were the most observed alterations. Areas of alveolar hyperinflation (Figure 3B,D) and vascular congestion (Figure 3D) were also observed. Furthermore, edema and focal areas of mild alveolar hemorrhage (Figure 3E and Figure 4F), also observed in the tissues of the infected mice, suggest that there was an alteration in vascular permeability.

**Figure 5:**
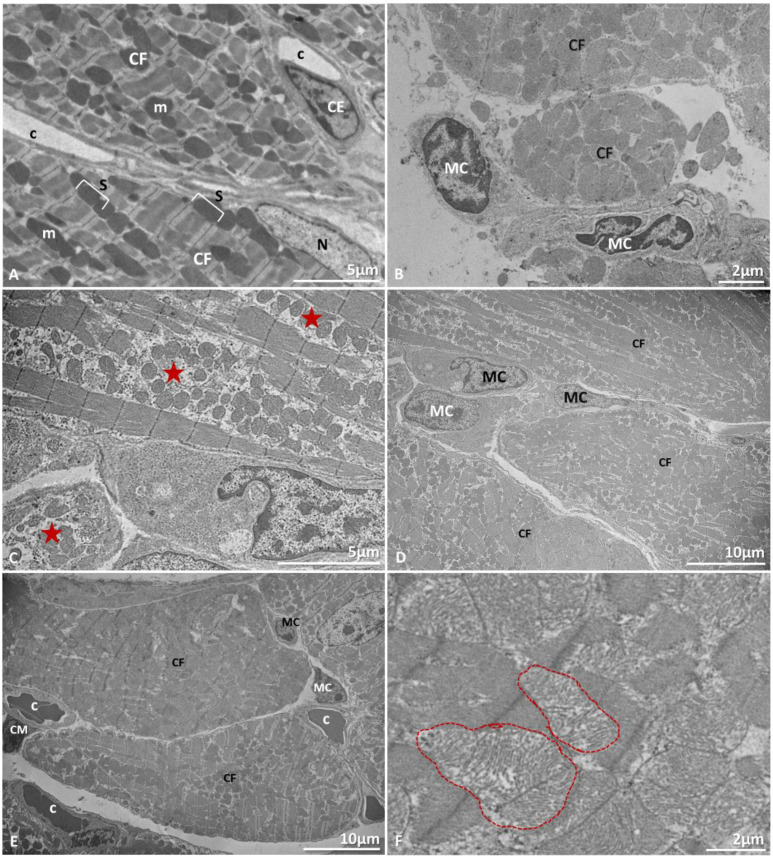
Ultrastructural alterations of heart of BALB/c mice. Euthanasia; 72 hpi. (**A**) Noninfected mouse. Cardiac fibers (CF), nucleus (N), mitochondrion (m), sarcomere (s), endothelial cell (CE), capillary (c). (**B**) Mononuclear cells (MC). (**C**) Degeneration of myofilaments (star). (**D**) mononuclear cells infiltration (MC). (**E**) Capillary congestion (C), mononuclear cells (MC). (**F**) Mitochondrial tumefaction (arrow). Experimental infection: (**B**) Lineage I, (**C**–**F**) Lineage II.

Heart tissues from noninfected BALB/c mice showed fusiform cardiomyocytes arranged in parallel bundles. Signs of edema, vascular congestion, hemorrhage and inflammatory cell infiltrate were not observed (Figure 4A and Figure 5A). The heart from BALB/c mice infected with both DENV-2 lineages showed fairly well-preserved areas. The histopathological changes observed in both groups were the presence of mostly mild inflammatory infiltrates (Figure 4C), although some samples presented an apparent increase in cellularity due to mononuclear infiltrates (Figure 4D) and apparent cytoplasmic rarefaction of cardiomyocytes (Figure 4B). Some of those cells presented lightly stained nuclei, as if some nuclear content was lost (Figure 4B).

Ultrastructural analysis of mice heart samples corroborated the histopathological findings. There were mononuclear cells infiltrate in the interstitium (Figure 5B,D,E) and areas of apparent loss of cytoplasmic content showed myofilament degeneration (Figure 5C). In addition, congested capillaries (Figure 5E) and alteration of mitochondria, characterized by apparent enlargement and a less electron dense appearance (Figure 5F), were observed. Table 2 shows the number of infected mice whose lungs or heart showed the aforementioned histopathological changes.

### 3.3. Morphometry

**Figure 6:**
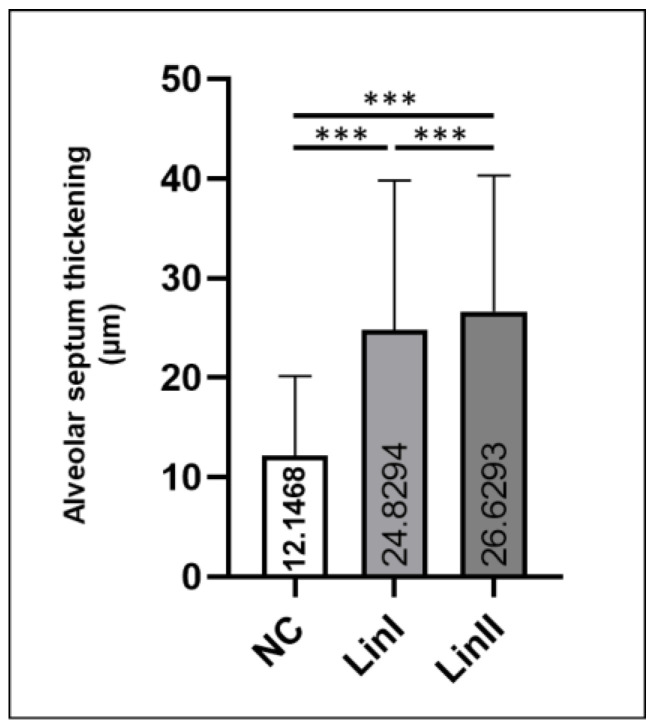
Morphometrical analysis of alveolar septum of infected and noninfected BALB/c mice. Euthanasia: 72 hpi. NC: negative control, Lin: lineage, hpi: hours post-infection, ***: *p* < 0.001.

In order to compare the magnitude of alveolar septum thickening in infected and control mice, the thickness of the structure was measured. Histomorphometric analysis showed that the mean thickening observed in lungs of mice infected with both DENV-2 strains (means = 24.82 µm and 26.61 µm, respectively) was greater than in the control group (mean = 12.14 µm). The difference between the three experimental groups was statistically significant (*p* < 0.001) (Figure 6).

### 3.4. Viral Genome and Antigen Detection

**Figure 7:**
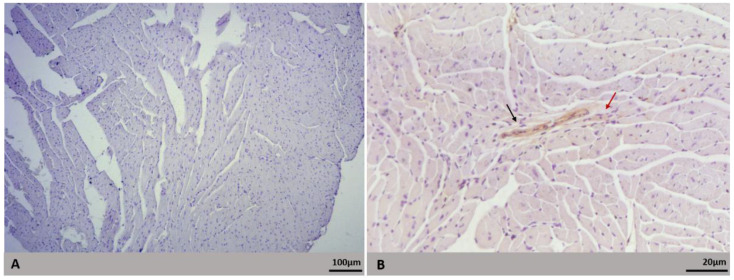
DENV antigen detection in heart of BALB/c mice infected with DENV-2 Lineage II. Euthanasia: 72 hpi. (**A**) Negative control showing no peroxidase reactive cells; (**B**) peroxidase-reactive endothelial cells (black arrow) and cardiomyocyte (red arrow).

Viral genome and antigen were not detected in lung samples. However, in mice infected with Lineage I, viral RNA was detected in three heart tissues (1.56 × 10^−1^; 6.7 × 10^3^ and 2.52 × 10^7^ RNA copies/µL) and two skeletal muscle tissues (1.59 × 10^0^ and 3.23 × 10^4^ RNA copies/µL). In mice infected with Lineage II, the viral genome was also detected in three heart tissue samples (3,58 × 10^0^, 3.79 × 10^7^ and 3.37 × 10^−2^ copies of RNA/µL) and two skeletal muscle samples (1.97 × 10^4^ and 2.7 × 10^2^ RNA copies/µL). Moreover, the viral antigen was detected in endothelial cells and cardiac fibers (Figure 7B) in one heart sample infected with Lineage II. Noninfected samples did not show peroxidase-reactive cells (Figure 7A).

### 3.5. Creatine Kinase (CK) Levels

**Figure 8:**
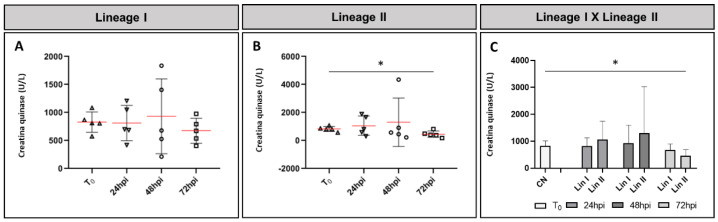
Creatine kinase (CK) levels present in serum from BALB/c mice noninfected (T_0_) and infected with DENV-2 strains I or II 24, 48 and 72 hpi. (**A**) Lineage I of DENV-2; (**B**) Lineage II of DENV-2; (**C**) comparison of mean creatine kinase levels. CN: negative control, Lin: lineage, hpi: hours post-infection, *: *p* ≤ 0.05.

The mean concentration of CK present in the serum of noninfected mice was 830 U/L. In mice infected with DENV-2 Lineage I, the average at 24 hpi, 813 U/L, was lower than the control group, but increased at 48 and 72 hpi, with averages of 933.2 U/L and 677.4 U/L, respectively. In mice infected with Lineage II, an increase in the enzyme level was observed 1 dpi and 2 dpi (means: 1059.4 U/L and 1307.4 U/L, respectively). At 72 hpi, the mean (459 U/L) observed was lower than in the control group. Figure 8 shows CK levels variation in serum samples from infected and noninfected mice. The difference between the means of the control group and the group infected with DENV-2 Lineage II was statistically significant at 72 hpi (*p* = 0.023).

## 4. Discussion

Dengue is an acute febrile disease caused by one of the four DENV serotypes, which cause similar clinical manifestations, although variations in intensity may occur due to the host genetic factors, heterophile immunity and infective strain characteristics [4,29,58]. Most patients are asymptomatic or develop non-severe dengue, presenting mild symptoms such as fever, headache, retro-orbital pain, gastrointestinal symptoms, myalgia, arthralgia and rash [59]. Bleeding manifestations include petechiae, ecchymosis, epistaxis, gingival hemorrhage and metrorrhagia, appearing at the end of the febrile period [60]. However, there are cases in which the disease can progress to severe dengue, a potentially lethal complication due to plasma leakage, fluid accumulation, respiratory distress, severe bleeding and multi-organ involvement [61].

The most frequently affected organ during DENV infection is the liver [6]. Nonetheless, DENV has been detected in different tissues [7,62,63], and neurological, renal, muscular, cardiac and pulmonary manifestations and disorders during severe dengue cases have been reported [8,9,23]. Unusual manifestations may be underreported and, since the majority of studies concern autopsy samples, data on different tissues involvement during non-severe cases of dengue are scarce [6,64,65,66]. Therefore, here, we aimed to evaluate alterations induced by the infection of two DENV-2 lineages in lung, heart and skeletal muscle using the immunocompetent BALB/c as an experimental model.

Pulmonary manifestations during dengue are not common [11]. Pleural effusion is a common finding in dengue patients who present with respiratory symptoms, and is the most frequent cause of dyspnea among patients. Ground-glass opacity and consolidation are seen less frequently and may represent pulmonary edema or hemorrhage [10,11,63,67,68]. A study that evaluated lung involvement during dengue using computed tomography scans reported that these changes are more frequent in severe dengue patients and suggested that this condition is related to plasma leakage [10]. In this study, an increase in lung weight in infected mice when compared to the control group was observed. Except for the Lineage I/14 dpi group, the means of infected animals were slightly higher. Regarding the lung weight/body weight ratio, the means of the infected groups were higher throughout the entire course of infection. Furthermore, the means of mice infected with Lineage II were higher than those infected with Lineage I, with a statistically significant difference at 14 dpi. Although there is a tendency of organ increase in infected mice, imaging exams were not performed in this study; therefore, it is not possible to assess whether the increase in lung weight was a result of pleural effusion. However, in our histological analysis, alveolar edema and hemorrhage were observed, as described in both human fatal cases and experimental models [6,10,47,69], and it would not be unjustified to associate such alterations with the mild increase in the average weight of this organ.

Few cases of severe pulmonary manifestation due to DENV infection have been reported [70,71,72], and a study carried out by Rodrigues et al. [10] showed that there is a tendency of mild to moderate lung involvement. Neither the genome nor the antigen of DENV was detected in our lung tissue samples. Nonetheless, there were alterations in the lungs of infected mice that were not present in noninfected ones. Here, we observed alveolar septum thickening and alveolar collapse due to the migration of inflammatory cells to the interstice. As a compensatory mechanism, some alveoli were hyperinflated. These finds are in accordance with investigation data of fatal cases and experimental model studies [6,10,38,40,44,47,69]. As mentioned above, some samples presented focal hemorrhage and edema, as seen in BALB/c mice infected with DENV-3 [47]. In human cases, lung hemorrhage is reported in fatal cases [6,10,69] and some patients with severe symptoms presented blood-tinged secretion coming from both lungs [71,72]. To our knowledge, no lung hemorrhage or edema concerning non-severe dengue have been reported in human cases, most likely due to the fact that pulmonary involvement is not investigated in such cases.

Although data on the cardiac involvement in dengue are scarce, lately, heart involvement has been increasingly reported [14,15,16,17,18] and it is mostly associated with severe cases [14,15,18]. Main findings include atrioventricular conduction disorders, supraventricular arrhythmias, myocarditis, bradycardia, tachycardia, pericardial effusion, diastolic dysfunction and increased levels of cardiac enzymes [4,16,18,73]. Cardiomegaly has been reported in cases of DENV infection [13,15,16,74] and it can be caused by coronary artery disease, hypertension, valvulopathies, pulmonary diseases and myocarditis, among other complications [75]. In this study, the mean heart weight and the heart weight/body weight ratio increased in infected mice. Only one group (Lineage II/72 h) presented a mean lower than the control group. In a study carried out with 10 patients with a positive diagnosis for arboviruses (dengue or chikungunya), nine had increased heart volume and another case study reported thickening of the ventricular wall [13,15]. Viral infections are the most common cause of myocarditis, which can result in dilated cardiomyopathy with consequent enlargement of the heart [16]. In this study, no apparent difference between the heart volume of infected and noninfected mice was observed. Histopathological analysis did not reveal edema or hemorrhage foci as having occurred in other organs; therefore, we cannot confirm that the accumulation of fluid in the interstitium resulted in an increase in the average weight of the heart, and further investigation must be carried out to better understand the causes of the weight change.

Most cardiac manifestations are attributed to myocarditis due to viral infection [9,16]. The presence of DENV in the heart has been demonstrated in fatal human cases [6,14,62,73]. In this study, the viral genome and antigen were detected in heart samples of mice infected with both lineages of DENV-2. Although it is not clear whether tissue damage is caused by direct viral infection or the host’s immune response [14,76], a number of histopathological alterations have been reported [6,64,73,76].

The tissues showed mild histopathological and ultrastructural alterations. Inflammatory infiltrates are commonly found in DENV-infected tissues and have been observed in both human cases and experimental models [6,64,73,76,77]. Miranda [73] reported that mononuclear infiltrate consisted mainly of CD68+ macrophage-type cells. Apparent cytoplasmic rarefaction and nuclear content loss of cardiomyocytes were observed in histological samples. Upon ultrastructural analysis, we could see that the rarefaction was a result of myofilaments degeneration, also seen by Miranda [64] and Póvoa [6]. Mitochondrial swelling was also observed. Póvoa [6] associated mitochondrial and nuclear alterations with apoptotic process of cardiac fibers. As observed by Caldas [47] and Rasinhas [44] in mice infected with DENV-3 and DENV-4, respectively, our samples showed small areas of vascular congestion. The changes present in our samples were moderate; however, studies carried out with BALB/c mice infected with serotypes 3 and 4 revealed changes in the morphology of intercalated discs, increases in heart rate due to reduced blood pressure and increased plasma leakage in the heart [44,47,51]. The differences observed in the alterations induced by different serotypes may indicate that a particular DENV serotype has greater or lesser tropism for a particular organ [78].

Myalgia and muscle weakness are manifestations presented by dengue patients. It has been reported that acute renal failure in dengue can be caused by rhabdomyolysis, a medical condition resulting in the dissolution of damaged or injured skeletal muscle [23,24,25,26,27]. Analysis of skeletal muscle biopsies from dengue patients revealed perivascular inflammatory infiltrate, mitochondrial proliferation, foci of necrosis with or without myofagocytosis, and interstitial hemorrhage [22,23,24,25]. The presence of edema, hemorrhage foci, and metabolic alterations may be responsible for transient muscle weakness [22]. In this study, morphological changes were not observed in the histological sections of the infected mice. However, viral RNA was detected in skeletal muscle samples from mice infected with both lineages of DENV-2. This finding is in agreement with a study of experimental infection of human myotubes culture that showed that DENV is able to infect and replicate in muscle cells [14].

Elevated CK levels are indicative of muscle, heart or brain damage. As CK-MB levels are more appropriate to assess cardiac damage, the most likely hypothesis is that the altered levels of the enzyme are due to muscle damage. On average, at 24 and 48 hpi, CK levels were higher in mice infected with both DENV-2 lineages, mainly in the Lineage II group. The individual analysis showed that six infected mice had CK levels well above the mean of each group (Lin I: 1209 U/L, 1837 U/L and 979 U/L and Lin II: 1897 U/L, 4354 U/L and 822 U/L). It is noteworthy that the mean CK level of the Lineage II group peaked at 48 hpi due to one individual (4354 U/L). This individual was not paired with the experimental group for histopathological studies, so tissue alteration could not be assessed. Our results are in agreement with studies of human dengue cases that report increases in CK levels. However, these studies also report functional and histopathological changes in skeletal muscle that were not observed here [22,23,26,27].

Dengue has a wide spectrum of clinical manifestations, with unpredictable evolution and involvement of different organs. Thus, the establishment of animal experimental models is of great relevance to provide a better understanding of pathogenesis mechanisms. BALB/c mice were shown to be an appropriate experimental model for dengue pathogenesis studies on lung, heart and skeletal muscle tissues, as we reported viral detection in heart and skeletal muscle and described tissue alterations in the lungs and hearts of mice infected with two distinct epidemic and non-adapted DENV-2 lineages.

## Figures and Tables

**Table 1:** Number of BALB/c used for histopathological, immunohistochemical and CK levels analysis, genome detection and measuring organs’ weight after experiments with Lineages I and II of the DENV-2 Asian/American genotype.

*N* = 123	Histopathology qRT-PCR/IHQ	TEM	CK Level Analysis			Organ Weight		
	72 hpi	72 hpi	24 hpi	48 hpi	72 hpi	72 hpi	7 dpi	14 dpi
DENV-2/Lineage I	10/10/5	5	5	5	5	22	15	15
DENV-2/Lineage II	10/10/5	5	5	5	5	22	15	15
Negative control	5/5/5	5			5			19
Total [samples]	25	15	35			123		

IHQ: immunohistochemistry, TEM: transmission electron microscopy, hpi: hours post-infection, dpi: days post-infection.

**Table 2:** Histopathological alterations observed in lung and heart samples of BALB/c infected with DENV-2 Lineages I or II and euthanized at 72 hpi. Number of mice whose livers presented the alteration/total number infected mice.

	Histopathological Alterations	DENV-2		
		Lineage I (%)	Lineage II (%)	Total (%)
Lung	Alveolar septum thickening	10/10 (100)	9/10 (90)	19/20 (95)
	Inflammatory infiltrate	10/10 (100)	9/10 (90)	19/20 (95)
	Vascular congestion	8/10 (80)	6/10 (60)	14/20 (70)
	Alveolar hemorrhage	6/10 (60)	6/10 (60)	12/20 (60)
	Edema	4/10 (40)	4/10 (40)	8/20 (40)
Heart	Inflammatory infiltrate	7/10 (70)	9/10 (90)	17/20 (85)
	Cytoplasmic rarefaction	7/10 (70)	4/10 (40)	11/20 (55)
	Increased cellularity	2/10 (20)	4/10 (40)	6/20 (30)

hpi: hours post-infection, dpi: days post-infection.

## Data Availability

Not applicable.
